# Vaccine cold chain in general practices: A prospective study in 75 refrigerators (Keep Cool study)

**DOI:** 10.1371/journal.pone.0224972

**Published:** 2019-11-19

**Authors:** Anika Thielmann, Marie-Therese Puth, Christine Kersting, Johannes Porz, Birgitta Weltermann

**Affiliations:** 1 Institute for General Practice, University of Duisburg-Essen, University Hospital Essen, Essen, Germany; 2 Institute of General Practice and Family Medicine, University of Bonn, Bonn, Germany; 3 Department of Medical Biometry, Informatics and Epidemiology, Faculty of Medicine, University of Bonn, Bonn, Germany; Food and Drug Administration, UNITED STATES

## Abstract

**Introduction:**

Protecting vaccines from freeze damage is considered one of the most poorly addressed problems in vaccine management. Freezing may impair the potency especially of adsorbed vaccines. The Keep Cool study aims at ensuring optimal vaccine storage conditions in general practices. This publication analyses the baseline data using standardised temperature recordings.

**Methods:**

This prospective study in German general practices analysed 7-day temperature recordings of refrigerators used for vaccine storage. Temperatures were recorded continuously using a standardised data logger with an accuracy of ±0.4 °C. The prevalence rates of refrigerators within the target range (2 to 8 °C) and of those reaching critically low temperatures (≤0 °C) were calculated. In addition, the cumulative time and the duration of single episodes beyond the target range were computed. To assess for structural deficits, the prevalence of refrigerators with a cycling of >5 °C was determined. Generalised linear mixed models were applied to analyse correlating factors between the dependent variables ‘within temperature range’ and ‘reaching critically low temperatures’ with practice characteristics.

**Results:**

The study included 64 of 168 practices (38.1% response rate) with 75 refrigerators. The prevalence of refrigerators with temperatures within the target range was 32.0% (n = 24), and 14.7% (n = 11) reached critically low temperatures <0 °C. 44.0% of refrigerators (n = 33) showed temperatures >8 °C and 28.0% (n = 21) <2 °C. Of the 168 hours recorded per refrigerator, the average cumulative time >8 °C was 49 hours, <2 °C 75 hours and ≤0 °C 74 hours. The longest consecutive period of critically low temperatures was 168 hours (mean: 39±53). The prevalence of refrigerators with a cycling range of >5 °C was 29.3%.

**Conclusion:**

Given the importance of immunisation, the results of our study call for action, as two-thirds of the refrigerators exhibited cold chain breaches and 15% reached critically low temperatures threatening vaccine potency.

## Introduction

Immunisations are among the most effective and cost-effective public health strategies worldwide [1]. However, their effectiveness depends on adequate vaccine storage conditions. Maintaining the cold chain, i.e. a temperature range of 2 °C to 8 °C, is crucial to ensure vaccine potency [2] and tolerability [3]. In the past, cold chain breaches were suspected of causing disease outbreaks, but confirming this suspicion is difficult [4–8]. Preventing freezing is especially important to maintain the potency of adsorbed vaccines (e. g. hepatitis A, hepatitis B, tetanus, diphtheria, pertussis, pneumococcal disease) [2]. Adsorbed hepatitis B vaccines are considered the most sensitive vaccines, with a freezing threshold of -0.5 °C [2]. At this temperature, irreversible precipitates of aluminium-containing adsorbents begin to form which decrease the potency of the vaccines. Also, these may induce local irritation upon injection [2,3]. Furthermore, all vaccines are at risk of contamination when exposed to freezing temperatures, as hairline cracks in the pre-filled syringe can develop which are not necessarily noticeable to the human eye [9]. The World Health Organization (WHO) considers protecting vaccines from freeze damage “one of the most poorly addressed problems in vaccine management” that requires attention in order not to jeopardise disease-prevention goals. [10].

Research of the American National Institute of Standards and Technology showed that refrigerators’ suitability to maintain the cold chain varies drastically depending on the type of refrigerator [11,12]. Relevant parameters are ‘temperature control stability, air circulation patterns, defrost cycles, and long-term drift of the temperature set point’ [11]. A crucial aspect is the technical design of the cooling compressor and its regulation based on on-off mechanisms. Purpose-built refrigerators for the storage of medical products, so-called pharmaceutical refrigerators, have several advantages compared to household models: enhanced temperature set point control, a better ventilation system, and a narrower temperature range [12]. Many household models are designed to allow different temperature zones required in food storage [13] and allow for freezing temperatures, e. g. -5 °C [11].

According to a systematic literature review, freezing temperature exposure occurred in approximately 33.3% of refrigerators used for vaccine storage in ten wealthier countries [14]. In our prior cross-sectional questionnaire study, 16% of German general practices self-reported experiencing cold chain breaches either as an error or near-error, and 49% lacked adequate monitoring and documentation [15,16]. The Keep Cool study aims at ensuring optimal vaccine storage conditions: after visual inspections of refrigerators used to store vaccines and a baseline temperature survey of seven days, general practices with temperature violations are offered access to a tailored online learning program [17,18].

This publication presents the baseline data of the Keep Cool study: standardised, continuous 7-day temperature data are analysed for cold chain breaches in general practices. First, we aimed to identify the prevalence of refrigerators with temperatures within the target range (2–8 °C). Second, we determined the prevalence of critically low temperatures (≤0 °C) and analysed the temperature cycling ranges of refrigerators in order to assess their capacity to maintain the cold chain. Third, associations between practice characteristics and temperature were analysed.

## Methods

### Study design

Details on this prospective intervention study with two temperature measurement periods have been reported elsewhere [17]. Briefly, this publication describes the baseline of the Keep Cool study, which was developed by two researchers (A.T., B.W.), formerly Institute for General Medicine, University of Duisburg-Essen, now: Institute for Family Medicine and General Practice, University of Bonn, Germany. We report about the quality of the vaccine cold chain in general practices with temperature readings over a 7-day monitoring period. Details on the quality of vaccine refrigerator management (e.g. temperature monitoring frequency, presence of a thermometer, placement of temperature probe) based on visual inspections of the refrigerators studied have been reported elsewhere [18].

Ethical approval was obtained from the Ethic Commission of the Medical Faculty of the University of Duisburg-Essen (14-6118-BO). Participants provided written informed consent.

### Study population and recruitment procedure

The study was conducted in general practices affiliated with the University of Duisburg-Essen (N = 185) as teaching practices. Practices (n = 17) involved in study pre-tests were excluded. Recruitment followed a structured approach: Practices were contacted by phone and fax up to three times or until they responded. To estimate participation bias, non-participants received a short questionnaire by fax asking them to provide details on: 1) their reason for non-participation, 2) the number of refrigerators in practices including those in the recreation room, 3) the use of a thermometer, and 4) whether the temperature is monitored twice daily.

### Temperature monitoring

Temperatures were measured with a data logger (testo 175T), which has an accuracy of ±0.4 °C within the operating range -5 °C to +10 °C (calibrated under a DIN EN ISO 9001:2008 certified quality assurance system). The device was equipped with a standard probe which measures the ambient air temperatures inside the refrigerator and the effects of door openings on refrigerator temperatures. We used continuous measurements over seven days with a logging interval of one reading per minute. Similar logging rates were used in prior studies [19–23].

**Fig 1:**
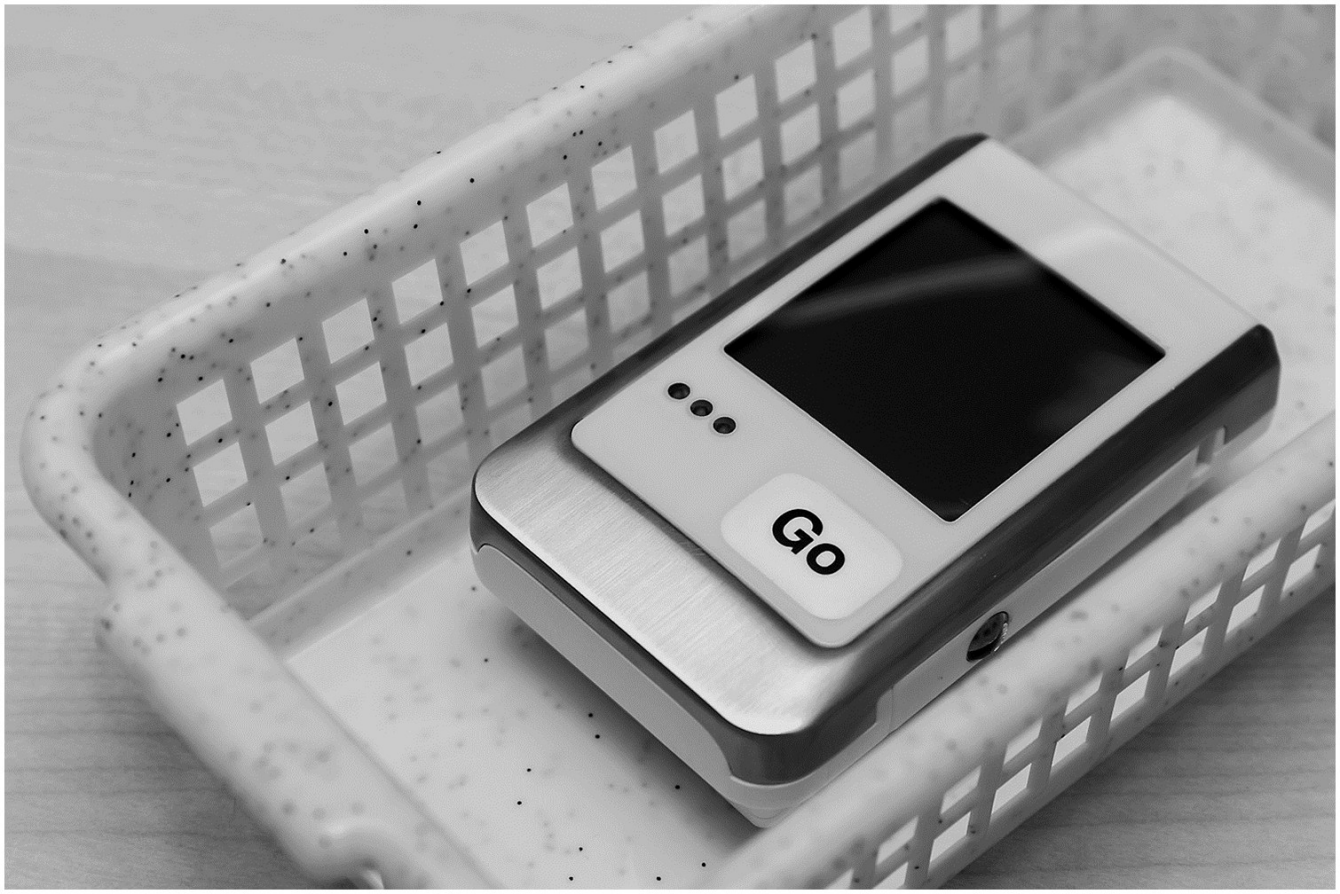
Set-up of data logger in plastic bin.

In preparation for this study, we developed a protocol for the set-up of the data logger which had been piloted in a sample of 17 general practices with 21 refrigerators. In line with standards [11–13,24], the data logger was positioned in the centre of the refrigerator and placed in a plastic bin (see Fig 1). During the recording, the display of the data logger was turned off and access to its memory was locked.

### Practice and physician characteristics

The following practice characteristics were obtained by questionnaire: type of practice (solo/group), number of practice team members by professional groups, patients per quarter (caseload), number of treatment rooms, thermometer in each vaccine refrigerator, vaccine spectrum offered, and selected services offered (tropical medicine and/or yellow fever, travel medicine, adolescent preventive services, paediatric preventive services and/or adolescent medicine), percentage of patients with statutory health insurance, and certified quality management. Data on the type of refrigerator used for vaccine storage were collected while setting up the data logger (pharmaceutical-grade/household refrigerator; location and insulation of ice compartment, if any).

### Statistical analysis

Descriptive statistics were used to describe participating and non-participating practices for practice and physician characteristics. For the non-participant analysis, the χ^2^ test was used for categorical data, and Student’s t-test was used for continuous data.

The temperature readings of the 7-day monitoring period (10,080 minutes) were analysed. The first 120 minutes after each data logger set-up were excluded from analyses to allow the probe to acclimatise to the temperature of the refrigerator.

The primary outcome was the prevalence of refrigerators with temperatures within the target range (2 °C to 8 °C) for seven days. Secondary outcomes included reaching different cut-offs based on data on temperature sensitivity of the WHO [2], personal manufacturer information (GlaxoSmithKline) and systematic reviews [14,25]: <2 °C and >8 °C, >8 °C, <2 °C, ≤1 °C, ≤0 °C. In order to provide a better indication of unacceptable temperature exposure, we calculated the cumulative and consecutive time (in hours) outside the target range and beyond different cut-offs. Analyses were performed for each individual refrigerator and for the total sample using mean, standard deviation (SD) and range.

A further secondary outcome addressed the refrigerators’ capacity to keep temperatures within the target range. A temperature cycling range >5.0 °C was considered unacceptable. Temperature ranges were analysed for each refrigerator, for the total sample and stratified by household and pharmaceutical-grade refrigerators as well as by refrigerators considered acceptable and unacceptable for vaccine storage. Acceptable refrigerators included pharmaceutical-grade, freezerless refrigerators and full-size dual-zone refrigerators/freezers with separate exterior doors, while unacceptable refrigerators included any mini refrigerators and refrigerators with an internal ice compartment [11–13].

To estimate the relationship between practice characteristics and the two dependent variables ‘within temperature range (2–8 °C) versus outside target range’ and ‘reaching critically low temperatures (0 °C) versus within target range’, we used hierarchical generalised linear mixed models (GLMM) for binomial responses with random practice-specific intercepts (to account for practices with more than one refrigerator). Independent characteristics were: type of practice (solo/group), number of patients in practice (≤1,750/ >1,750), percentage of patients with statutory health insurance, yellow fever licence (yes/no), physician trainee in practice (yes/no), certified quality management (yes/no), and the provision of paediatric preventive services and/or adolescent medicine (yes/no).

Statistical analyses were performed using IBM SPSS Statistics for Windows, version 24 (Armonk, NY: IBM Corp.) and R, version 3.5.1. Percentages and mean values are reported for valid cases.

The trial is registered with the German Clinical Trials Register (DRKS00006561).

## Results

### Practice characteristics

Of the 168 practices contacted, 64 agreed to participate (response rate: 38.1%). The mean practice size was 2.1 general practitioners (±1.2) and 5.3 medical assistants (±3.3); 59.4% (n = 38) were group practices. 51.9% (n = 27) of the practices provided medical care to up to 1,750 patients per quarter (caseload). In total, 75 refrigerators were included in this study. 14.1% (n = 9) had more than one refrigerator for the storage of vaccines. See Table 1 for details.

**Table 1:** Characteristics of participating practices (N = 64).

	n	%*
**Practice type**		
Solo	26	40.6
Group	38	59.4
**Staff**		
Mean no. of physicians in practice ± SD [10]	2.1±1.2	
Mean no. of medical assistants ± SD [11]	5.3±3.3	
**Number of treatment rooms** [10]		
≤ 3	31	57.4
> 3	23	42.6
**Patients per practice per quarter (caseload)** [12]		
≤ 1,750	27	51.9
> 1,750	25	48.1
**Percentage of patients with statutory health insurance** [8]		
≤ 85%	19	33.9
> 85%	37	66.1
**Certified quality management** [15]	14	28.6
**Physician qualifications**		
Travel medicine [10]	12	22.2
Tropical medicine and/or yellow fever license [10]	7	13.0
**Services offered** [10]		
Paediatric preventive services and/or adolescent medicine	22	40.7
Adolescent preventive services	44	81.5
**Practice vaccine spectrum** [10]		
Mean no. of vaccines ±SD	17.9±1.8	
Tetanus	54	100.0
Diphtheria	54	100.0
Pertussis	54	100.0
Influenza	54	100.0
Pneumococcal disease	54	100.0
Hepatitis A	54	100.0
Measles	54	100.0
Poliomyelitis	53	98.1
Hepatitis B	53	98.1
Tick-borne encephalitis	53	98.1
Rubella	53	98.1
Mumps	53	98.1
Meningococcal disease	52	96.3
Typhus	50	92.6
Varicella	50	92.6
Rabies	46	85.2
Human papilloma	44	81.5
Haemophilus influenzae b	41	75.9
Cholera	26	48.1
Rotavirus	14	25.9
**Refrigerator type** (n = 75)		
Pharmaceutical grade	9	12.0
Household model	66	88.0
Freezerless refrigerator	30	47.0
Refrigerator with internal ice compartment (one exterior door)	31	45.5
Refrigerator with internal non-insulated ice compartment (one exterior door)	2	3.0
Full-size dual-zone refrigerator/freezer (separate exterior doors)	2	3.0
Unclear	1	1.5

*valid percentages

[missing values]

73 of the 104 non-participating practices provided a reason for non-participation. The most frequent reasons were (multiple responses): no time (37.0%, n = 27), no interest in topic/study participation (35.6%, n = 26), no need (11.0%, n = 8), other (∑7, 23.3%, n = 17). The non-participant analysis showed no differences regarding key practice characteristics, except that participating practices were more likely to provide care to ≤1,750 patients per quarter (48.1% versus 68.1%, p = 0.029). See S1 Table for details.

### Refrigerators and temperature recordings

**Fig 2:**
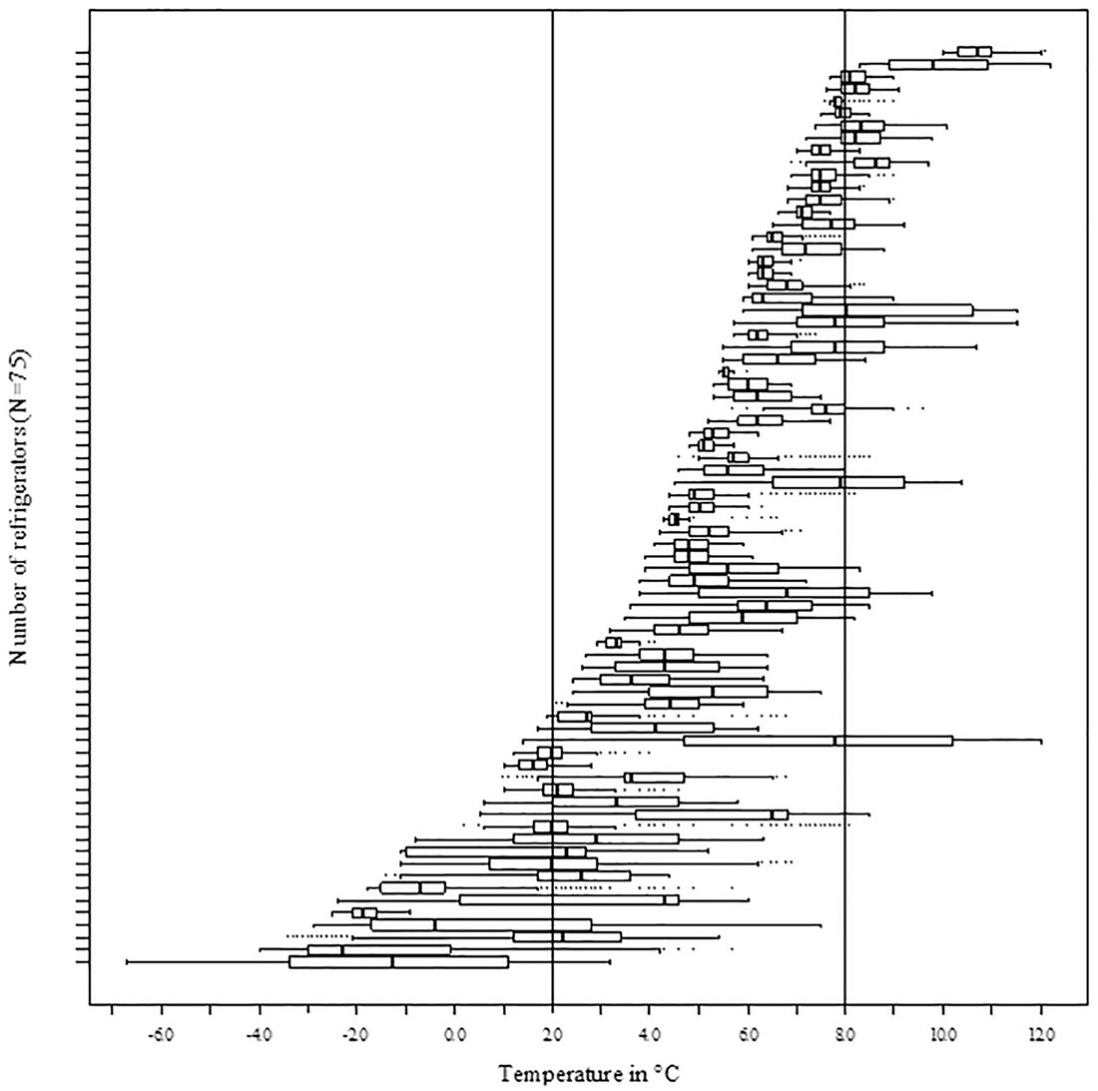
Temperature ranges per refrigerator (N = 75).

Of the 75 refrigerators included, 88.0% (n = 66) were household refrigerators and 12.0% (n = 9) were pharmaceutical-grade models (see Table 1 for details). In 24 of 75 included refrigerators (32.0%), temperatures were within the target range of 2 °C to 8 °C (see Fig 2). This corresponds to 74.8% of the total measurement time. The mean temperature was 5.3 °C (±2.9), with readings ranging between -6.7 and +12.2 °C.

Based on their ability to maintain the target temperature range, refrigerators were categorised into six exclusive groups (Table 2). Refrigerators that were within the target range but had at least one temperature breach >8 °C (n = 28) had a mean temperature of 7.3 °C (±0.7) and were outside the target range 25.3% of the time. Refrigerators within the target range that had at least one temperature breach <2 °C (n = 17) had a mean temperature of 1.8 °C (±1.5) and were outside the target range 45.5% of the time. Separate data on individual refrigerators is presented in S2 Table.

**Table 2:** Refrigerators categorised according to their ability to maintain the target temperature range, i.e. 2 to 8 °C (N = 75).

	No. of refrigerators (n = 75)		Target range		Temperature			
	n	%	% inside	% outside	mean±SD on average	min-max of all means	mean of all ranges	min-max of all ranges
Within target range but at least once >8 °C	28	37.3	74.7	25.3	7.3±0.7	5.1–8.8	3.5	1.3–6.0
Always within target range	24	32.0	100.0	0.0	5.2±0.5	3.3–6.6	2.4	0.7–5.1
Within target range but at least once <2 °C	17	22.7	54.5	45.5	1.8±1.5	-1.2–4.0	6.5	1.8–10.4
Always >8 °C	2	2.7	0.0	100.0	10.3±0.8	9.9–10.7	3.0	2.1–3.9
<2 °, in target range, >8 °C	3	4.0	64.4	35.6	5.0±2.0	2.0–7.5	8.8	7.9–10.6
Always <2 °C	1	1.3	0.0	100.0	-1.8±0.4	n/a	2.6	n/a

Mean and SD refer to row n

Critically low temperatures ≤0 °C were recorded at least once in 14.7% (n = 11) of all refrigerators. This corresponds to 5.8% of the temperature recording time, i.e. the total time based on all refrigerators (mean cumulative time: 74.1 hours ±56.1). The longest consecutive time ≤0 °C was 39.1 hours on average (±52.9: 0.6–168.0): one refrigerator was below zero at all times (168 hours), for the other refrigerators the average cumulative time was 64.7 hours). Temperatures <2 °C were recorded in 28.0% (n = 21) of refrigerators, corresponding to 12.4% of the complete temperature recording time. These refrigerators had a mean of 31.6 episodes (±36.3: 1–142) below <2 °C with an average duration of 18.7 hours (±41.0: 12 min to 168 hours). Further temperature cut-offs are presented in Table 3. On average, the target temperature range (8 °C) was exceeded for 48.6 hours (±51.4). This accounts for 12.7% of the total study. See Table 3 for details.

**Table 3:** Overview of temperature recordings based on different cut-offs (N = 75).

Cut-offs	No. of refrigerators (n = 75)^+^		Cumulative time (in hours)			No. of episodes		Duration of episodes (in hours)		Longest consecutive time (in hours)	
	n	%	mean±SD on average	study total*	% of study total^#^	mean±SD on average	min-max of all means	mean±SD on average	min-max of all means	mean±SD on average	min-max of all means
**Above target range**											
>8 °C	33	44.0	48.6±51.4	1,605.4	12.7	39.5± 42.3	1–145	11.3±40.4	0.1–168.0	17.7±41.9	0.1–168.0
**Below target range**											
<2 °C	21	28.0	74.5±52.3	1,565.5	12.4	31.6±36.3	1–142	18.7±41.0	0.2–168.0	34.1±50.9	0.3–168.0
≤1 °C	17	22.7	57.7±58.8	981.7	7.8	16.6±17.9	1–55	19.5±42.9	0.1–168.0	27.2±46.7	0.1–168.0
≤0 °C	11	14.7	74.1±56.1	815.1	6.5	14.7±15.8	1–45	28.8±51.4	0.4–168.0	39.1±52.9	0.6–168.0
≤-0.5 °C	11	14.7	74.1±55.4	729.8	5.8	12.2±13.2	1–45	27.8±50.8	0.2–166.1	36.4±52.9	0.3–166.1
≤-3.0 °C	3	4.0	33.5±28.6	100.4	0.8	20.7±21.4	2–44	1.5±1.4	0.3–3.0	4.8±5.6	0.5–11.1

All values refer to row n

^+^Of the 51 refrigerators with temperatures beyond the target range, n = 3 had temperatures below and above the target range. For this reason, numbers do not add up to 100%.

^#^Refers to the percentage of the study total time based on 75*168h.

### Structural characteristics: Cycling range of refrigerators

**Fig 3:**
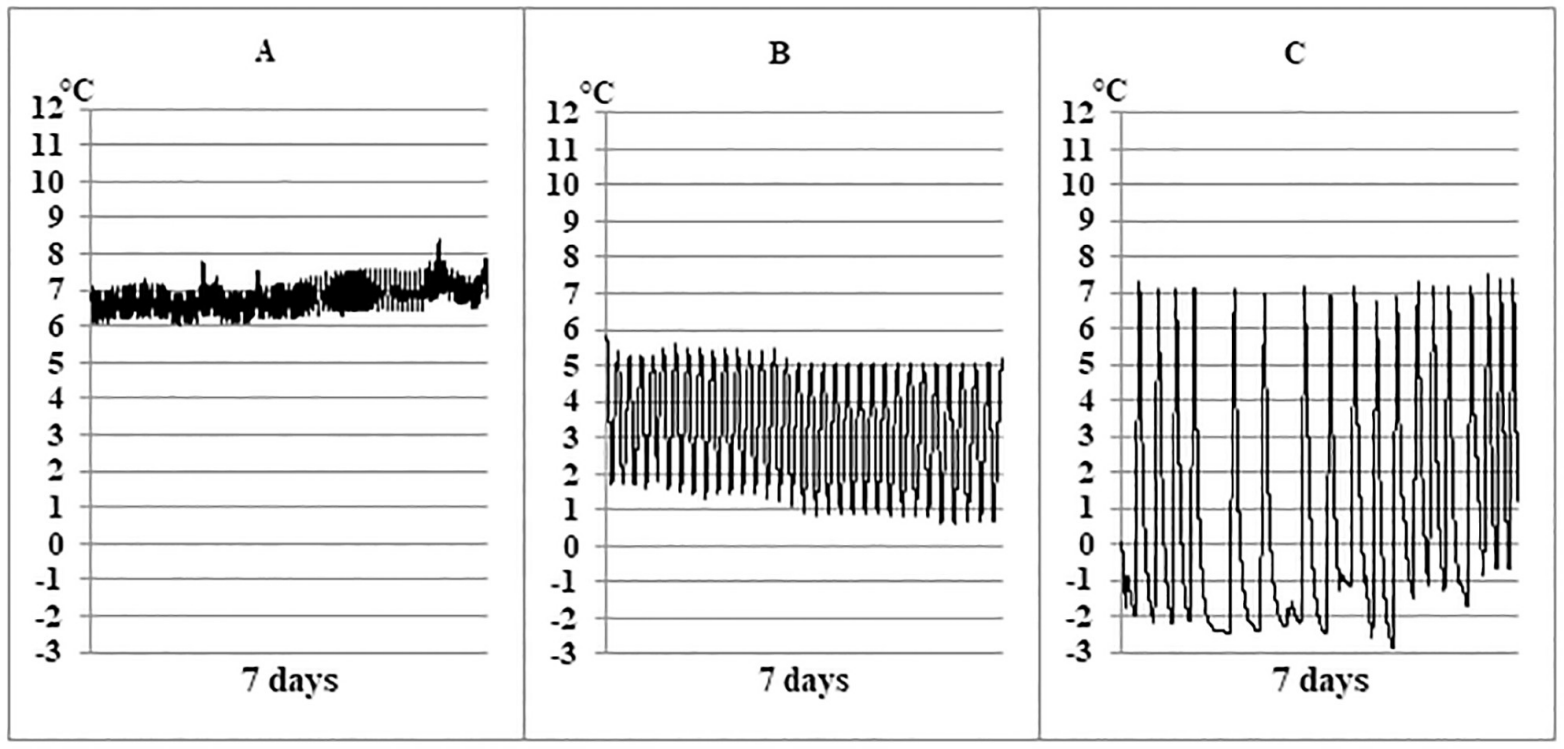
Examples of temperature recordings in three different refrigerators. All data were recorded at 1-minute intervals over 7 days. The graphs illustrate common problems regarding temperature cycling range and mean temperature: A: an adequate cycling range of 3°C with a too-high mean temperature at approx. 7°C; B: a cycling range of 5.5°C with a too-low mean temperature at approx. 3.5°C; C: a too-high cycling range of 10°C and a too-low mean temperature at approx. 2°C.

In 29.3% (n = 22) of refrigerators, temperature cycling was >5 °C. Cycling ranges ≤3.0 °C were recorded in 45.3% (n = 34), >3.0 to ≤4.0 °C in 17.3% (n = 13), >4 °C in 37.3% (n = 28) and >6 °C in 17.3% (n = 13). Of the 22 refrigerators with cycling ranges >5 °C, only one refrigerator maintained the cold chain. In comparison, in refrigerators with cycling ranges ≤5 °C, 43.4% (n = 23) maintained the cold chain and 56.6% (n = 30) had cold chain breaches. Refrigerators varied with regard to the range of temperature cycling during the day and over the course of the 7-day monitoring period. Fig 3 shows typical temperature curves encountered. The overall mean temperature range was 4.0 °C (±2.5), with readings ranging between 0.7 to 10.6 °C.

The mean temperature in pharmaceutical refrigerators (n = 9) was 5.3 °C (±1.1: 4.3 to 7.9 °C) with a mean temperature range of 3.1 (±1.5: 0.7 to 5.9 °C). In comparison, the mean temperature in household refrigerators (n = 66) was 5.3 °C (±2.8: -1.8 to 10.7 °C) with a mean temperature range of 4.1 (±2.6: 1.0 to 10.6 °C).

### Associations between temperature outcomes and practice characteristics

Analysis using the GLMM showed no significant associations between both dependent variables (within temperature range, critically low temperature) and the independent variables considered.

## Discussion

Of the 75 refrigerators analysed, only 32% maintained the vaccine cold chain. However, 68% were beyond the target range and 15% reached a critically low temperature of 0 °C. We found that continuous freezing temperature exposure lasted longer than one day on average (39 hours) with a longest episode of seven days recorded. These data suggest that freeze damage likely occurred.

In line with the systematic review of freezing temperatures by Hanson [14] to assess whether freezing remains an ongoing issue, we cannot answer the question regarding the number of vaccines that were actually damaged or had reduced immunogenicity. Nevertheless, there is a link between disease outbreak and temperature excursions below the freezing threshold for hepatitis B [26] and pertussis [4]. In our practice sample, more than 98% store hepatitis B vaccines. Thus, until thermostable vaccines are available or freeze-free technologies are used across all practices, freeze prevention requires close attention.

To ensure the cold chain, two components need to be fulfilled: 1) structural component with a cycling range below 5 °C and 2) continuous refrigerator management targeting for a mean temperature of +5 °C. In 29% of refrigerators, cycling ranges exceeded the cut-off of 5 °C and thus constituted a major barrier for successful cold-chain maintenance. Interestingly, even in refrigerators with narrower temperature ranges, about 60% failed to maintain the cold chain, indicating procedural deficits.

In line with prior studies, our observations in the practices during the study conduct shed an interesting light on key influencing factors. Practices did not have adequate temperature monitoring rigor (i. e., a suitable thermometer, monitoring at least twice daily), which is a significant predictor of noticing critical temperatures [18,25]. Overall, knowledge and problem awareness deficits prevailed, which is known from other countries [19–21,27]. For instance, physicians and medical assistants expressed astonishment with regard to the encountered temperature ranges when shown the temperature curves of their refrigerators. With the exception of door opening times, most participants expected rather stable temperatures, as they were unaware of the construction-based cycling of refrigerators. In practices affected by freezing temperature exposure, we even encountered disbelief (‘your thermometer is broken’). There was a general belief that freezing temperatures would be noticed in the form of frozen vaccines. Frequently, constant fluctuations between freezing and thawing were never considered before. Misshapen cardboard packaging due to thawing ice (two practices) and thick ice walls (one practice) went unnoticed (for details see Thielmann et al.) [18].

### Limitation

All participating practices are members of a teaching practice network. A potential selection bias can be excluded as we showed in a prior study that the practice sample is representative for general practices in Germany [28]. In order to assess participation bias, we conducted a thorough non-responder analysis. For financial reasons, we used a standard air probe to measure temperature. In contrast to that, a slow-reacting glycol probe resembles the temperature changes of the vaccine vials. Given the accuracy, all measured temperature values are within ±0.4 °C of the true value.

### Conclusion

The prevalence of refrigerators with cold chain breaches and critically low temperatures is high, which emphasises the need for an intervention aimed at adequate vaccine storage. Risk communication should address the dangers associated with too cold temperatures and refrigerators’ temperature cycling issues. Furthermore, greater attention needs to be paid to structural and procedural best practices in vaccine storage that are used as a safeguard against temperature excursions.

## Supporting information

S1 Table Characteristics of participating practices (N = 64). (DOCX)

S2 Table Temperature recordings per refrigerator (N = 75). (DOCX)
