# Supplementary material for: Vaccine cold chain in general practices: A prospective study in 75 refrigerators (Keep Cool study)
Source: PLoS One. 2019 Nov 19;14(11):e0224972. doi: 10.1371/journal.pone.0224972 (PMC6863523; doi:10.1371/journal.pone.0224972)
Supplement: S1 Table — (DOCX) [file pone.0224972.s001.docx]

**S1 Table. Non-participant analysis (n=168).**

|  | | | |  | |  |  |  | | |  | | | **Participants**  **(n=64)** | | | | | | **Non-participants**  **(n=104)** | | | | | **p-value** | |
| --- | --- | --- | --- | --- | --- | --- | --- | --- | --- | --- | --- | --- | --- | --- | --- | --- | --- | --- | --- | --- | --- | --- | --- | --- | --- | --- |
|  | | | |  | | | | | | | | | | **n** | | | **%** | |  | **n** | | **%** | |  |  | |
| **Practice type** | | | |  | | | | | | | | | |  | | |  | |  |  | |  | |  |  | |
|  | Solo | | | | | | | | | | | | | 26 | | | 40.6 | |  | 39 | | 37.5 | |  | 0.810 | |
|  | Group | | | | | | | | | | | | | 38 | | | 59.4 | |  | 65 | | 62.5 | |  |  | |
| **Patients per practice per quarter (caseload)** | | | | | | | | | | | | | |  | | |  | |  |  | |  |  | |  |  |
|  | ≤ 1,750 | | | | | | | | | | | | | 27 | | | 51.9 | |  | 29 | | 31.9 | |  | 0.029 | |
|  | > 1,750 | | | | | | | | | | | | | 25 | | | 48.1 | |  | 62 | | 68.1 | |  |  | |
| **Mean percentage of patients with statutory health insurance** | | | | | | | | | |  | | |  | | | 87.3±10.0 | | |  |  | 83.8±16.0 | | |  | 0.150 | |
| **Staff** | | | |  | | | | | | | | | |  | |  | | |  |  |  | | |  |  | |
|  | Mean no. of physicians in practice | | | | | | | | | | | | |  | | 2.1±1.2 | | |  |  | 2.3±1.3 | | |  | 0.234 | |
|  | Mean no. of medical assistants | | | | | | | | | | | | |  | | 5.3±3.3 | | |  |  | 5.1±2.9 | | |  | 0.792 | |
| **Certified quality management** | | | |  | | | | | | | | | |  | | |  | |  |  | |  | |  |  | |
|  | | No | | | | | | | | | | | | 35 | | | 71.4 | |  | 53 | | 66.3 | |  | 0.676 | |
|  | | Yes | | | | | | | | | | | | 14 | | | 28.6 | |  | 27 | | 33.8 | |  |  | |
| **Tropical medicine and/or yellow fever license** | | | | | | | | |  | | | | |  | | |  | |  |  | |  | |  |  | |
|  | | No | | | | | | | | | | | | 47 | | | 87.0 | |  | 75 | | 82.4 | |  | 0.616 | |
|  | | Yes | | | | | | | | | | | | 7 | | | 13.0 | |  | 16 | | 17.6 | |  |  | |
| **Services offered** | | | | |  | | | | | | | | |  | | |  | |  |  | |  | |  |  | |
|  | Paediatric preventive services and/or adolescent medicine | | | | | | | | | | | | |  | | |  | |  |  | |  | |  |  | |
|  | | | No | | | | | | | | | | | 32 | | | 59.3 | |  | 66 | | 72.5 | |  | 0.142 | |
|  | | | Yes | | | | | | | | | | | 22 | | | 40.7 | |  | 25 | | 27.5 | |  |  | |
|  | Adolescent preventive services | | | | | | | | | | | | |  | | |  | |  |  | |  | |  |  | |
|  | | | No | | | | | | | | | | | 10 | | | 18.5 | |  | 15 | | 16.5 | |  | 0.932 | |
|  | | | Yes | | | | | | | | | | | 44 | | | 81.5 | |  | 76 | | 83.5 | |  |  | |
| **Practice vaccine spectrum** | | | | | | | | | | | | |  | | |  | |  |  |  |  | | |  |  | |
|  | Mean no. of vaccines (range) | | | | | | | | | | | |  | | | 17.9 ±1.8 | | |  |  | 17.0±2.5 | | |  | 0.017 | |
| **Thermometer in each vaccine refrigerator** | | | | | | | | | | | |  | | |  | |  | |  |  | |  | |  |  | |
|  | | No | | | | | | | | | | | | | 0 | | 0.0 | |  | 4 | | 4.1 | |  | 0.297* | |
|  | | Yes | | | | | | | | | | | | | 54 | | 100.0 | |  | 93 | | 95.9 | |  |  | |

^*^Fisher’s exact test

Missing values: participants/non-participants: practice type: 0/0; caseload: 12/13; patients with statutory health insurance: 8/28; staff: physicians 10/12; staff: medical assistants 11/15; certified quality management: 15/24; tropical medicine and/or yellow fever license: 10/13; services offered 10/13; no. of vaccines: 10/12; thermometer in each vaccine refrigerator: 10/7.
