# Supplementary material for: Vaccine cold chain in general practices: A prospective study in 75 refrigerators (Keep Cool study)
Source: PLoS One. 2019 Nov 19;14(11):e0224972. doi: 10.1371/journal.pone.0224972 (PMC6863523; doi:10.1371/journal.pone.0224972)
Supplement: S2 Table — (DOCX) [file pone.0224972.s002.docx]

**S2 Table. Temperature recordings per refrigerator (n=75)**

| **Refrigera-tor no.** | **Type of refrigerator** | **Mean** | **Min** | **Max** | **Range** | **SD** | **% in target range (≥2 to ≤8 °C)** | **≤0 °C** | | | | **<2 °C** | | | | **>8 °C** | | | |
| --- | --- | --- | --- | --- | --- | --- | --- | --- | --- | --- | --- | --- | --- | --- | --- | --- | --- | --- | --- |
|  |  |  |  |  |  |  |  | **%** | **Longest consecutive time (in hours) ≤0 °C** | **Cumulative time (in hours) ≤0 °C** | **No. of episodes ≤0 °C** | **%** | **Longest consecutive time (in hours) <2 °C** | **Cumulative time (in hours) <2 °C** | **No. of episodes <2 °C** | **%** | **Longest consecutive time (in hours) >8 °C** | **Cumulative time (in hours) >8 °C** | **No. of episodes >8 °C** |
| 1 | Refrigerator with internal ice compartment | 5.2 | 2.4 | 7.5 | 5.1 | 1.4 | 100.0 |  |  |  |  |  |  |  |  |  |  |  |  |
| 2 | Refrigerator with internal ice compartment | 4.0 | 1.7 | 6.2 | 4.5 | 1.3 | 96.5 |  |  |  |  | 3.6 | 0.3 | 6.0 | 27 |  |  |  |  |
| 3 | Refrigerator with internal ice compartment | 2.6 | -1.4 | 4.4 | 5.8 | 1.1 | 68.3 | 0.8 | 1.4 | 1.4 | 1 | 31.7 | 2.6 | 53.2 | 58 |  |  |  |  |
| 4 | Refrigerator with internal ice compartment | 7.7 | 6.5 | 9.2 | 2.7 | 0.6 | 67.7 |  |  |  |  |  |  |  |  | 32.3 | 0.9 | 54.2 | 103 |
| 5 | Freezerless refrigerator | 8.3 | 7.4 | 10.1 | 2.7 | 0.5 | 36.5 |  |  |  |  |  |  |  |  | 63.5 | 9.3 | 106.6 | 92 |
| 6 | Freezerless refrigerator | 5.9 | 3.5 | 8.2 | 4.7 | 1.2 | 99.8 |  |  |  |  |  |  |  |  | 0.2 | 0.3 | 0.4 | 2 |
| 7 | Freezerless refrigerator | 7.6 | 6.9 | 9.0 | 2.1 | 0.4 | 88.8 |  |  |  |  |  |  |  |  | 11.2 | 8.8 | 18.8 | 44 |
| 8 | Freezerless refrigerator | 5.7 | 3.9 | 8.3 | 4.4 | 1.0 | 99.9 |  |  |  |  |  |  |  |  | 0.1 | 0.2 | 0.2 | 1 |
| 9 | Refrigerator with internal non-insulated ice compartment | 4.4 | 2.0 | 5.9 | 3.9 | 0.7 | 100.0 |  |  |  |  |  |  |  |  |  |  |  |  |
| 10 | Freezerless refrigerator | 6.8 | 3.8 | 9.8 | 6.0 | 1.8 | 68.2 |  |  |  |  |  |  |  |  | 31.8 | 1.4 | 53.5 | 43 |
| 11 | Household model unclear | 7.7 | 6.8 | 9.2 | 2.4 | 0.6 | 79.2 |  |  |  |  |  |  |  |  | 20.8 | 24.4 | 35.0 | 22 |
| 12 | Freezerless refrigerator | 6.8 | 6.0 | 8.4 | 2.4 | 0.4 | 99.7 |  |  |  |  |  |  |  |  | 0.3 | 0.5 | 0.5 | 1 |
| 13 | Freezerless refrigerator | 5.2 | 4.8 | 5.8 | 1.0 | 0.2 | 100.0 |  |  |  |  |  |  |  |  |  |  |  |  |
| 14 | Refrigerator with internal ice compartment | 4.4 | 2.6 | 6.4 | 3.8 | 1.1 | 100.0 |  |  |  |  |  |  |  |  |  |  |  |  |
| 15 | Pharmaceutical grade | 5.5 | 5.4 | 6.1 | 0.7 | 0.1 | 100.0 |  |  |  |  |  |  |  |  |  |  |  |  |
| 16 | Refrigerator with internal ice compartment | 2.0 | -1.1 | 6.9 | 8.0 | 1.9 | 51.3 | 20.0 | 33.5 | 33.6 | 2 | 48.7 | 53.4 | 81.9 | 4 |  |  |  |  |
| 17 | Refrigerator with internal ice compartment | 2.1 | 1.0 | 4.7 | 3.7 | 0.4 | 63.4 |  |  |  |  | 36.6 | 4.3 | 61.5 | 142 |  |  |  |  |
| 18 | Refrigerator with internal ice compartment | 3.3 | 0.6 | 5.8 | 5.2 | 1.4 | 75.7 |  |  |  |  | 24.3 | 1.8 | 40.8 | 32 |  |  |  |  |
| 19 | Refrigerator with internal ice compartment | 6.2 | 5.2 | 7.7 | 2.5 | 0.5 | 100.0 |  |  |  |  |  |  |  |  |  |  |  |  |
| 20 | Freezerless refrigerator | 7.3 | 6.6 | 11.7 | 5.1 | 0.8 | 96.1 |  |  |  |  |  |  |  |  | 3.9 | 6.6 | 6.6 | 1 |
| 21 | Freezerless refrigerator | 7.9 | 7.6 | 9.2 | 1.6 | 0.2 | 93.2 |  |  |  |  |  |  |  |  | 6.8 | 3.7 | 11.4 | 6 |
| 22 | Refrigerator with internal ice compartment | 5.7 | 4.6 | 8.0 | 3.4 | 0.7 | 100.0 |  |  |  |  |  |  |  |  |  |  |  |  |
| 23 | Freezerless refrigerator | 6.2 | 5.7 | 7.4 | 1.7 | 0.2 | 100.0 |  |  |  |  |  |  |  |  |  |  |  |  |
| 24 | Refrigerator with internal ice compartment | 6.4 | 6.0 | 7.0 | 1.0 | 0.2 | 100.0 |  |  |  |  |  |  |  |  |  |  |  |  |
| 25 | Freezerless refrigerator | 6.3 | 6.0 | 7.1 | 1.1 | 0.2 | 100.0 |  |  |  |  |  |  |  |  |  |  |  |  |
| 26 | Refrigerator with internal ice compartment | -1.8 | -2.5 | 0.1 | 2.6 | 0.4 | 0 | 100.0 | 168.0 | 168.0 | 1 | 100.0 | 168.0 | 168.0 | 1 |  |  |  |  |
| 27 | Refrigerator with internal ice compartment | 7.3 | 6.1 | 8.8 | 2.7 | 0.6 | 82.5 |  |  |  |  |  |  |  |  | 17.5 | 1.0 | 29.4 | 69 |
| 28 | Freezerless refrigerator | 3.7 | 2.4 | 6.3 | 3.9 | 0.8 | 100.0 |  |  |  |  |  |  |  |  |  |  |  |  |
| 29 | Refrigerator with internal ice compartment | 5.3 | 4.8 | 6.2 | 1.4 | 0.3 | 100.0 |  |  |  |  |  |  |  |  |  |  |  |  |
| 30 | Refrigerator with internal ice compartment | 6.3 | 5.3 | 7.5 | 2.2 | 0.6 | 100.0 |  |  |  |  |  |  |  |  |  |  |  |  |
| 31 | Freezerless refrigerator | 7.5 | 5.2 | 9.6 | 4.4 | 0.7 | 75.7 |  |  |  |  |  |  |  |  | 24.3 | 1.3 | 40.8 | 145 |
| 32 | Refrigerator with internal ice compartment | 6.0 | 5.3 | 6.9 | 1.6 | 0.4 | 100.0 |  |  |  |  |  |  |  |  |  |  |  |  |
| 33 | Refrigerator with internal ice compartment | 8.6 | 6.9 | 9.7 | 2.8 | 0.5 | 13.4 |  |  |  |  |  |  |  |  | 86.6 | 50.8 | 145.5 | 52 |
| 34 | Freezerless refrigerator | -1.2 | -6.7 | 3.2 | 9.9 | 2.5 | 16.3 | 63.7 | 4.0 | 107.1 | 45 | 83.7 | 4.7 | 140.6 | 45 |  |  |  |  |
| 35 | Freezerless refrigerator | 7.6 | 7.0 | 9.2 | 2.2 | 0.3 | 94.4 |  |  |  |  |  |  |  |  | 5.6 | 2.3 | 9.4 | 24 |
| 36 | Freezerless refrigerator | 6.7 | 5.5 | 8.4 | 2.9 | 0.8 | 96.6 |  |  |  |  |  |  |  |  | 3.4 | 0.3 | 5.7 | 64 |
| 37 | Refrigerator with internal ice compartment | 5.2 | 4.2 | 7.1 | 2.9 | 0.5 | 100.0 |  |  |  |  |  |  |  |  |  |  |  |  |
| 38 | Freezerless refrigerator | 7.8 | 4.5 | 10.4 | 5.9 | 1.5 | 53.5 |  |  |  |  |  |  |  |  | 46.4 | 2.1 | 78.0 | 44 |
| 39 | Freezerless refrigerator | 5.0 | 4.4 | 6.4 | 2.0 | 0.4 | 100.0 |  |  |  |  |  |  |  |  |  |  |  |  |
| 40 | Refrigerator with internal ice compartment | 2.1 | 1.2 | 4.0 | 2.8 | 0.7 | 56.1 |  |  |  |  | 43.9 | 38.7 | 73.7 | 27 |  |  |  |  |
| 41 | Freezerless refrigerator | 8.3 | 7.2 | 10.6 | 3.4 | 0.6 | 38.7 |  |  |  |  |  |  |  |  | 61.3 | 14.7 | 103.0 | 130 |
| 42 | Refrigerator with internal ice compartment | 8.2 | 7.7 | 9.0 | 1.3 | 0.3 | 41.2 |  |  |  |  |  |  |  |  | 58.8 | 5.5 | 98.7 | 115 |
| 43 | Refrigerator with internal ice compartment | 2.9 | -0.8 | 6.3 | 7.1 | 1.9 | 63.8 | 9.2 | 0.6 | 15.4 | 40 | 36.3 | 1.3 | 60.9 | 59 |  |  |  |  |
| 44 | Freezerless refrigerator | 5.0 | 3.8 | 7.2 | 3.4 | 0.6 | 100.0 |  |  |  |  |  |  |  |  |  |  |  |  |
| 45 | Freezerless refrigerator | 7.8 | 5.5 | 10.7 | 5.2 | 1.1 | 56.3 |  |  |  |  |  |  |  |  | 43.7 | 2.8 | 73.4 | 44 |
| 46 | Freezerless refrigerator | 9.9 | 8.3 | 12.2 | 3.9 | 1.1 | 0 |  |  |  |  |  |  |  |  | 100.0 | 168.0 | 168.0 | 1 |
| 47 | Freezerless refrigerator | 8.8 | 5.9 | 11.5 | 5.6 | 1.8 | 52.6 |  |  |  |  |  |  |  |  | 47.4 | 77.9 | 79.6 | 16 |
| 48 | Freezerless refrigerator | 1.1 | -1.1 | 5.2 | 6.3 | 1.9 | 54.9 | 44.9 | 75.5 | 75.5 | 1 | 45.1 | 75.7 | 75.7 | 1 |  |  |  |  |
| 49 | Refrigerator with internal ice compartment | 2.6 | 1.9 | 6.8 | 4.9 | 0.6 | 94.9 |  |  |  |  | 5.1 | 4.2 | 8.6 | 16 |  |  |  |  |
| 50 | Refrigerator with internal ice compartment | 2.1 | -3.5 | 5.4 | 8.9 | 1.7 | 54.4 | 11.0 | 2.2 | 18.4 | 13 | 45.6 | 3.0 | 76.7 | 76 |  |  |  |  |
| 51 | Freezerless refrigerator | 7.5 | 1.4 | 12 | 10.6 | 3.0 | 51.6 |  |  |  |  | 0.8 | 0.5 | 1.4 | 5 | 47.5 | 2.3 | 79.9 | 36 |
| 52 | Pharmaceutical grade | 4.5 | 4.3 | 6.6 | 2.3 | 0.2 | 100.0 |  |  |  |  |  |  |  |  |  |  |  |  |
| 53 | Refrigerator with internal ice compartment | 0.7 | -2.9 | 7.5 | 10.4 | 2.9 | 30.5 | 54.5 | 13.2 | 91.6 | 18 | 69.5 | 14.6 | 116.8 | 19 |  |  |  |  |
| 54 | Freezerless refrigerator | -1.2 | -4.0 | 5.9 | 9.9 | 2.6 | 16.4 | 75.8 | 26.0 | 127.3 | 13 | 83.6 | 27.0 | 140.5 | 13 |  |  |  |  |
| 55 | Pharmaceutical grade | 4.8 | 4.1 | 5.9 | 1.8 | 0.4 | 100.0 |  |  |  |  |  |  |  |  |  |  |  |  |
| 56 | Pharmaceutical grade | 5.8 | 4.6 | 8.6 | 4.0 | 0.4 | 99.6 |  |  |  |  |  |  |  |  | 0.4 | 0.6 | 0.6 | 1 |
| 57 | Refrigerator with internal ice compartment | 5.4 | 0.5 | 8.5 | 8.0 | 2.1 | 87.2 |  |  |  |  | 12.5 | 1.5 | 21.0 | 16 | 0.3 | 0.6 | 0.6 | 1 |
| 58 | Freezerless refrigerator | 6.5 | 6.1 | 7.9 | 1.8 | 0.3 | 100.0 |  |  |  |  |  |  |  |  |  |  |  |  |
| 59 | Freezerless refrigerator | 8.2 | 7.6 | 9.1 | 1.5 | 0.4 | 39.1 |  |  |  |  |  |  |  |  | 60.9 | 5.3 | 102.4 | 98 |
| 60 | Refrigerator with internal ice compartment | 8.0 | 7.5 | 9.4 | 1.9 | 0.3 | 69.7 |  |  |  |  |  |  |  |  | 30.3 | 17.4 | 51.0 | 51 |
| 61 | Refrigerator with internal ice compartment | 6.6 | 5.9 | 9.0 | 3.1 | 0.6 | 96.9 |  |  |  |  |  |  |  |  | 3.1 | 3.2 | 5.3 | 2 |
| 62 | Pharmaceutical grade | 4.8 | 3.9 | 6.1 | 2.2 | 0.4 | 100.0 |  |  |  |  |  |  |  |  |  |  |  |  |
| 63 | Freezerless refrigerator | 6.5 | 3.6 | 8.5 | 4.9 | 1.0 | 98.1 |  |  |  |  |  |  |  |  | 1.9 | 0.2 | 3.2 | 33 |
| 64 | Refrigerator with internal ice compartment | 3.9 | 1.0 | 6.8 | 5.8 | 0.9 | 98.3 |  |  |  |  | 1.7 | 2.8 | 2.9 | 2 |  |  |  |  |
| 65 | Refrigerator with internal non-insulated ice compartment | 1.6 | 1.0 | 2.8 | 1.8 | 0.4 | 18.9 |  |  |  |  | 81.1 | 103.7 | 136.2 | 92 |  |  |  |  |
| 66 | Freezerless refrigerator | 3.3 | 2.9 | 4.1 | 1.2 | 0.2 | 100.0 |  |  |  |  |  |  |  |  |  |  |  |  |
| 67 | Full-size dual-zone refrigerator/freezer (separate exterior doors) | 10.7 | 10.0 | 12.1 | 2.1 | 0.4 | 0 |  |  |  |  |  |  |  |  | 100.0 | 168.0 | 168.0 | 1 |
| 68 | Refrigerator with internal ice compartment | 7.5 | 6.8 | 8.4 | 1.6 | 0.2 | 99.8 |  |  |  |  |  |  |  |  | 0.2 | 0.3 | 0.4 | 2 |
| 69 | Refrigerator with internal ice compartment | 2.0 | 0.2 | 8.1 | 7.9 | 0.7 | 54.4 |  |  |  |  | 45.6 | 23.4 | 76.6 | 22 | 0 | 0.1 | 0.1 | 1 |
| 70 | Pharmaceutical grade | 5.1 | 4.4 | 8.2 | 3.8 | 0.5 | 99.8 |  |  |  |  |  |  |  |  | 0.2 | 0.3 | 0.3 | 1 |
| 71 | Pharmaceutical grade | 7.9 | 5.7 | 11.6 | 5.9 | 1.1 | 55.2 |  |  |  |  |  |  |  |  | 44.8 | 3.1 | 75.2 | 58 |
| 72 | Pharmaceutical grade | 4.6 | 3.2 | 6.7 | 3.5 | 0.6 | 100.0 |  |  |  |  |  |  |  |  |  |  |  |  |
| 73 | Full-size dual-zone refrigerator/freezer (separate exterior doors) | 2.6 | -2.4 | 6.0 | 8.4 | 2.5 | 61.8 | 24.4 | 11.9 | 41.0 | 24 | 38.2 | 26.3 | 64.2 | 5 |  |  |  |  |
| 74 | Refrigerator with internal ice compartment | -0.5 | -1.8 | 5.9 | 7.7 | 1.4 | 5.6 | 81.0 | 93.6 | 136.0 | 4 | 94.4 | 157.6 | 158.6 | 2 |  |  |  |  |
| 75 | Pharmaceutical grade | 4.2 | 2.7 | 6.4 | 3.7 | 0.7 | 100.0 |  |  |  |  |  |  |  |  |  |  |  |  |
